# Joint Resource Optimization for Orthogonal Frequency Division Multiplexing Based Cognitive Amplify and Forward Relaying Networks

**DOI:** 10.3390/s20072074

**Published:** 2020-04-07

**Authors:** Dong Qin, Tianqing Zhou

**Affiliations:** 1 School of Information Engineering, Nanchang University, Nanchang 330031, China; 2 School of Information Engineering, East China Jiaotong University, Nanchang 330013, China; zhoutian930@163.com

**Keywords:** cognitive radio, amplify and forward, OFDM

## Abstract

This paper investigates two resource allocation problems in cognitive relaying networks where both secondary network and primary network coexist in the same frequency band and adopt orthogonal frequency division multiplexing (OFDM) technology. The first one is the sum rate maximization problem of a secondary network under total power budget of a secondary network and tolerable interference constraint of a primary network. The second one is the sum rate maximization problem of a secondary network under separate power budgets of a secondary network and tolerable interference constraint of a primary network. These two optimization problems are completely different from those in traditional cooperative communication due to interference management constraint condition. A joint optimization algorithm is proposed, where power allocation and subcarrier pairing are decomposed into two subproblems with reasonable cost. The first one is a closed form solution of power allocation of the secondary network while managing the interference to a primary network under a constraint condition. The other is optimal subcarrier pairing at given power allocation. Simulation results reveal aspects of average signal to noise ratio (SNR), interference level, relay position, and power ratio on the sum rate of a secondary network.

## 1. Introduction

Cognitive radio technology can effectively alleviate the tension of spectrum resources and improve spectrum utilization because it allows secondary users to opportunistically access and simultaneously share spectrum resources by spectrum hole technology. In the meantime, relaying technology has shown its great potential to expand the cell coverage radius and save energy in long distance communications. Therefore, the introduction of cognitive radio into the relay network can further help the secondary user to increase its throughput.

For instance, the false alarm and missed detection events were considered in [1] in two-way cognitive cooperative networks, where secondary users accessed the spectrum in a hybrid interweave underlay way. Incorporating these practical issues into the hybrid cognitive networks, a suboptimal power allocation strategy for the aim of sum rate maximization and outage probability minimization of the secondary users was proposed in [1]. Unlike rate mission in most works, an outage probability target was developed in [2] in cognitive two-way relay networks, where a power allocation scheme was proposed to meet a qualify of service constraint of the primary users without channel state information. A probabilistic admission control of the primary users and a randomized service of the secondary users were designed in [3] in cooperative cognitive radio networks, where the average delay of the secondary users was optimized. The authors proved the equivalence of throughput maximization and delay minimization and developed a line search method. Different from traditional power allocation in previous works, the authors in [4] chose the number of power levels as optimization variables to define the throughput of secondary users. An energy efficiency problem was studied in [5] in cognitive relay networks with energy harvesting, where the secondary user was allowed to collect energy from the primary user. The goal of [6] was to obtain an approximate symbol error probability in a multi-antenna relay system. However, the direct link between a source and a relay was absent. Although a direct link was included in [7], power allocation was absent due to the simplicity in the process of deriving approximate symbol error probability.

Incorporation of orthogonal frequency division multiplexing (OFDM) technology in broadband communications can alleviate multipath fading against fluctuating channel conditions. In [8], the optimal power allocation, subcarrier pairing, and relay selection matrix were found in OFDM cognitive two-way relay networks with imperfect spectrum sensing. However, the authors in [8] operated a discrete searching method instead of a closed form solution to find optimal power due to the complex rate formula in the presence of imperfect spectrum sensing. In addition, the step size needs to be adjusted according to the actual implement to ensure the power precision. A similar sum rate maximization problem of secondary users was considered in [9] in OFDM based two-way cognitive relaying networks, where a per subcarrier interference constraint was introduced to protect the primary users. A full duplex relaying mode was provided in [10], where a secondary relay established a two-way link for a pair of primary users. As a reward, this full duplex relay station acted as a secondary user and transmitted its own messages with the remaining OFDM subcarriers. This mutually beneficial cooperation not only satisfied the requirements of primary users, but also benefited secondary users. A carrier aggregation technology was investigated in [11] to overcome spectrum limitation challenges. Different frequency bands were treated differently: low frequency band for direct link and high frequency band for dual-hop link. A bit error rate constraint was incorporated in [12] in OFDM based cooperative cognitive radio networks, where the secondary user adapted its constellation size according to the distance to primary user. The heterogeneous genetic algorithm was adopted in [13] to resolve a non convex problem in cognitive decode and forward (DF) relay networks. An interference control problem was proposed in [14,15] in cognitive radio networks, but they were both based on traditional point to point communication instead of cooperative communication. In [16], the system capacity was optimized under the constraints of total power and interference level. However, no cooperative technology was used in [16].

This paper considers cognitive amplify and forward (AF) relay networks operating in OFDM scheme. A concise comparison between previous works and our paper is shown in Table 1. The differences between our paper and previous works mainly lie in three aspects. Firstly, different links: previous works often only considered either relaying link [6,17] or only direct link [7], so there was no problem in choosing which link for the source to convey messages. However, the relaying link is not always better than the direct link and vice versa due to the fluctuating channel quality. In our paper, both relaying link and direct link are included and the selection criteria are provided according to the strength of both links. Secondly, different goals: although some work involves both links, their goal is to calculate accurate performance formulas rather than resource optimization [12,18,19]. However, what we are concerned about is the joint optimization of power allocation and subcarrier pairing. Thirdly, different system models: different from most works [18,19,20,21,22], where a primary user selected a secondary user acting as a relay to help the primary user forward the message. However, in our model, the secondary user doesn’t participate in the communication process of the primary user as long as the secondary user meets the interference management condition.

Through the above analysis, the cognitive radio technology based on relay assistance has not been fully explored. In this paper, we design a joint optimization algorithm in the cognitive relaying networks, where the secondary user can switch between direct link and relaying link depending on the channel conditions while maintaining an acceptable interference size at the primary user. Two types of typical problems are studied. One is to look for sum rate maximum of secondary network subject to total power budget of secondary network and tolerable interference level of primary network. The other one is to seek for sum rate maximum of secondary network subject to individual power budget of secondary network and tolerable interference level of primary network. In order to find the maximum value, we propose a joint optimization algorithm, in which power allocation and subcarrier pairing are decomposed into two subproblems. The first step is to find a closed form solution of power allocation without violating the interference constraint of the primary network. In particular, when the direct link is active, the power allocation reduces to a classical water-filling form. Then, the subcarrier pairing is found when the power has been solved at the first step.

## 2. System Model

Consider a relay aided cognitive network where a secondary source user SS establishes a link with a secondary destination user SD via a secondary AF relay station SR. Unlike [17], where the direct path from SS to SD is ignored, we consider a more general scenario with a direct path. The primary network consists of a pair of primary source user PS and primary destination user PD. The secondary users share the whole spectrum with the primary users in underlay mode. Assume that the spectrum is divided into *N* subcarriers in OFDM mode. Denote the channel coefficients from SS to SR and to SD over subcarrier *i* as $h_{s,i}$ and $h_{d,i}$, respectively. Similarly, the channel coefficient from SR to SD over subcarrier *j* is represented by $h_{r,j}$. The subcarrier pairing technique is applied here. Subcarrier *i* of the received signal and subcarrier *j* of the forwarded signal form a subcarrier pair $\left(i,j\right)$. Then, ${\tilde{h}}_{s,i}$ and ${\tilde{h}}_{r,j}$ denote the interference channel coefficients of SS→PD link and SR→PD link, respectively. It is well known that interference is mutual. The equivalent channel coefficients from PS to SR and from PS to SD are denoted by $h_{s,i}^{p}$ and $h_{d,i}^{p}$, respectively.

The transmission powers at SS and SR are given by $p_{s,i}$ and $p_{r,j}$, respectively. Similarly, the transmission power at PS is denoted by $q_{s,i}$. The data rate of the secondary user over the subcarrier pair $\left(i,j\right)$ is given by

(1) $$\begin{array}{cc} r_{i,j}= & \frac{1}{2}\log_{2}\left(1+p_{s,i}g_{i}+\frac{p_{s,i}a_{i}p_{r,j}b_{j}}{p_{s,i}a_{i}+p_{r,j}b_{j}+1}\right)\approx \frac{1}{2}\log_{2}\left(1+p_{s,i}g_{i}+\frac{p_{s,i}a_{i}p_{r,j}b_{j}}{p_{s,i}a_{i}+p_{r,j}b_{j}}\right) \end{array}$$

where

(2) $$a_{i}=\frac{{\left|h_{s,i}\right|}^{2}}{q_{s,i}{\left|h_{s,i}^{p}\right|}^{2}+N_{0}},b_{j}=\frac{{\left|h_{r,j}\right|}^{2}}{q_{s,j}{\left|h_{d,j}^{p}\right|}^{2}+N_{0}},g_{i}=\frac{{\left|h_{d,i}\right|}^{2}}{q_{s,i}{\left|h_{d,i}^{p}\right|}^{2}+N_{0}}$$

and $N_{0}$ is noise variance. The approximation in Equation (1) has been widely applied, such as [17].

## 3. Resource Allocation under Total Power Constraint

Although joint optimization of physical layer, medium layer, and application layer can achieve better performance [23], the successful establishment of communication depends first on the connection of the physical layer. Hence, this section focuses on the optimization of the physical layer. In this section, we prepare to seek for the maximum value of the sum rate of the secondary network subject to the available power budget and the tolerable interference threshold at the primary user. This problem is formulated by

(3) $$\begin{array}{cc} \max_{\left\{p_{s,i},p_{r,j},\rho_{i,j}\right\}} & \sum_{i=1}^{N}\sum_{j=1}^{N}\rho_{i,j}r_{i,j}\\ s.t. & \sum_{i=1}^{N}\sum_{j=1}^{N}\left(p_{s,i}+p_{r,j}\right)\leq P_{T}\\ \; & \sum_{i=1}^{N}p_{s,i}c_{i}\leq Q_{1},\sum_{j=1}^{N}p_{r,j}d_{j}\leq Q_{2}\\ \; & \sum_{i=1}^{N}\rho_{i,j}=1,\sum_{j=1}^{N}\rho_{i,j}=1,\forall i,j \end{array}$$

where $c_{i}={\left|{\tilde{h}}_{s,i}\right|}^{2}/N_{0}$, $d_{j}={\left|{\tilde{h}}_{d,j}\right|}^{2}/N_{0}$, $P_{T}$ is the total power budget at the secondary network, $Q_{1}$ and $Q_{2}$ denote the tolerable interference threshold that PD can maintain at the first phase and the second phase, respectively. $\rho_{i,j}\in \left\{1,0\right\}$ is a binary variable indicating whether the subcarrier pairing $\left(i,j\right)$ is successful. If subcarrier pairing is formed, then $\rho_{i,j}=1$; otherwise, $\rho_{i,j}=0$. Although our problem is a mixed integer programming problem due to the existence of discrete variables, the dual gap is asymptotically zero according to the time sharing property of OFDM [24].

### 3.1. Power Allocation

The Lagrangian in Equation (3) is given by

(4) $$\begin{array}{cc} L & =\sum_{i=1}^{N}\sum_{j=1}^{N}\rho_{i,j}r_{i,j}+\lambda_{1}\left[P_{T}-\sum_{i=1}^{N}\sum_{j=1}^{N}\rho_{i,j}\left(p_{s,i}+p_{r,j}\right)\right]+\mu_{1}\left[Q_{1}-\sum_{i=1}^{N}p_{s,i}c_{i}\right]+\mu_{2}\left[Q_{2}-\sum_{j=1}^{N}p_{r,j}d_{j}\right]\\ & =\sum_{i=1}^{N}\sum_{j=1}^{N}\rho_{i,j}\left[r_{i,j}-\lambda_{1}\left(p_{s,i}+p_{r,j}\right)-\mu_{1}p_{s,i}c_{i}-\mu_{2}p_{r,j}d_{j}\right]+\lambda_{1}P_{T}+\mu_{1}Q_{1}+\mu_{2}Q_{2} \end{array}$$

where $\lambda_{1}$, $\mu_{1}$ and $\mu_{2}$ are dual variables associated with the power and interference constraints. According to the Karush–Kuhn–Tucker criterion [25], we take a partial derivative on *L* with respect to $p_{s,i}$ and $p_{r,j}$ as

(5) $$\begin{array}{cc} \frac{\partial L}{\partial p_{s,i}}= & \frac{a_{i}b_{j}^{2}p_{r,j}^{2}+a_{i}^{2}g_{i}p_{s,i}^{2}+b_{j}^{2}g_{i}p_{r,j}^{2}+2a_{i}b_{j}g_{i}p_{s,i}p_{r,j}}{2\ln2\left(a_{i}p_{s,i}+b_{j}p_{r,j}\right)\left(a_{i}p_{s,i}+b_{j}p_{r,j}+a_{i}b_{j}p_{s,i}p_{r,j}+b_{j}g_{i}p_{s,i}p_{r,j}+a_{i}g_{i}p_{s,i}^{2}\right)}-\lambda_{1}-\mu_{1}c_{i} \end{array}$$

(6) $$\begin{array}{cc} \frac{\partial L}{\partial p_{r,j}}= & \frac{a_{i}^{2}b_{j}p_{s,i}^{2}}{2\ln2\left(a_{i}p_{s,i}+b_{j}p_{r,j}\right)\left(a_{i}p_{s,i}+b_{j}p_{r,j}+a_{i}b_{j}p_{s,i}p_{r,j}+b_{j}g_{i}p_{s,i}p_{r,j}+a_{i}g_{i}p_{s,i}^{2}\right)}-\lambda_{1}-\mu_{2}d_{j} \end{array}$$

Setting the partial derivative Equations (5) and (6) to be zero, we get the optimal power allocation given by

(7) $$p_{s,i}^{⋆}={\left[\frac{a_{i}b_{j}^{2}f_{i,j}^{2}+a_{i}^{2}g_{i}+2a_{i}b_{j}g_{i}f_{i,j}+b_{j}^{2}g_{i}f_{i,j}^{2}}{2\ln2\left(a_{i}+b_{j}f_{i,j}\right)\left(\lambda_{1}+c_{i}\mu_{1}\right)\left(b_{j}g_{i}f_{i,j}+a_{i}b_{j}f_{i,j}+a_{i}g_{i}\right)}-\frac{a_{i}+b_{j}f_{i,j}}{b_{j}g_{i}f_{i,j}+a_{i}b_{j}f_{i,j}+a_{i}g_{i}}\right]}^{+}$$

(8) $$p_{r,j}^{⋆}=f_{i,j}p_{s,i}$$

where

(9) $$f_{i,j}=\left\{\begin{array}{cc} \frac{a_{i}\sqrt{\left(\lambda_{1}+d_{j}\mu_{2}\right)\left(a_{i}b_{j}\left(\lambda_{1}+c_{i}\mu_{1}\right)+b_{j}g_{i}\left(\lambda_{1}+c_{i}\mu_{1}\right)-a_{i}g_{i}\left(\lambda_{1}+d_{j}\mu_{2}\right)\right)}-a_{i}g_{i}\left(\lambda_{1}+d_{j}\mu_{2}\right)}{b_{j}\left(a_{i}+g_{i}\right)\left(\lambda_{1}+d_{j}\mu_{2}\right)} & \mathrm{if}b_{j}\left(\lambda_{1}+c_{i}\mu_{1}\right)\\ \; & >g_{i}\left(\lambda_{1}+d_{j}\mu_{2}\right)\\ 0 & \mathrm{otherwise} \end{array}\right.$$

and ${\left(\cdot \right)}^{+}=\max\left(0,\cdot \right)$. Different from the standard water-filling form, the powers in Equations (7) and (8) are tailored for the interference management constraints by interference channel gains $c_{i}$ and $d_{j}$. As can be seen from the formula Equation (9), $f_{i,j}$ is the criterion for choosing the relaying link and the direct link. If the condition $b_{j}\left(\lambda_{1}+c_{i}\mu_{1}\right)>g_{i}\left(\lambda_{1}+d_{j}\mu_{2}\right)$ holds, the relaying link is more advantageous than the direct link. Otherwise, SS would prefer to choose a direct link rather than the aid from SR. In particular, in the case of direct transmission, power allocation degenerates into a classical water filling algorithm given by

(10) $$p_{s,i}^{⋆}={\left[\frac{1}{2\ln2\left(\lambda_{1}+c_{i}\mu_{1}\right)}-\frac{1}{g_{i}}\right]}^{+}$$

and $p_{r,j}=0$.

Next, let’s look at an interesting special case. When there is only one subcarrier (*N* = 1), corresponding to a narrow band flat fading scenario, there are four different cases of optimal power allocation.

(1) If the interference threshold of the primary network is large enough to tolerate the power value at which the secondary network achieves its maximum rate without interference constraint, then the optimal power allocation is exactly the same as that without interference constraint. Mathematically, the optimal power allocation is given by

(11) $$p_{s}^{\left(1\right)}=\left\{\begin{array}{cc} \frac{b\left(g+\sqrt{ab-ag+bg}\right)P_{T}}{\sqrt{ab-ag+bg}\left(b+\sqrt{ab-ag+bg}\right)} & \mathrm{if}b>g\\ P_{T} & \mathrm{otherwise} \end{array}\right.$$

(12) $$p_{r}^{\left(1\right)}=\left\{\begin{array}{cc} \frac{a\left(b-g\right)P_{T}}{\sqrt{ab-ag+bg}\left(b+\sqrt{ab-ag+bg}\right)} & \mathrm{if}b>g\\ 0 & \mathrm{otherwise} \end{array}\right.$$

if $Q_{1}/c\geq p_{s}^{\left(1\right)}$ and $Q_{2}/d\geq p_{r}^{\left(1\right)}$ hold. Note the superscript indicates the first case and the subcarrier index subscript is omitted for brevity due to N=1.

(2) If the interference threshold of the primary network is less than the power constraint of the secondary network, then the power allocation is determined entirely by the interference threshold. In this case, the power allocation is given by $p_{s}^{\left(2\right)}=Q_{1}/c$ and $p_{r}^{\left(2\right)}=Q_{2}/d$ if $Q_{1}/c+Q_{2}/d<P_{T}$ holds.

(3) If the interference threshold is too small for the secondary source while too large for the secondary relay, then the power allocation is constrained by the source’s interference threshold. Under this circumstance, the power allocation is given by $p_{s}^{\left(3\right)}=Q_{1}/c$ and $p_{r}^{\left(3\right)}=P_{T}-Q_{1}/c$ if $Q_{1}/c+Q_{2}/d>P_{T}$ and $Q_{1}/c<p_{s}^{\left(1\right)}$ hold.

(4) If the interference threshold is too large for the secondary source while too small for the secondary relay, then the power allocation is constrained by the relay’s interference threshold. Under this circumstance, the power allocation is given by $p_{s}^{\left(4\right)}=P_{T}-Q_{2}/d$, and $p_{r}^{\left(4\right)}=Q_{2}/d$ if $Q_{1}/c+Q_{2}/d>P_{T}$ and $Q_{2}/d<p_{r}^{\left(1\right)}$ hold.

### 3.2. Subcarrier Pairing

By substituting the optimal power allocation into Equation (4), the dual function becomes

(13) $$L=\sum_{i=1}^{N}\sum_{j=1}^{N}\rho_{i,j}\varphi_{i,j}+\lambda_{1}P_{T}+\mu_{1}Q_{1}+\mu_{2}Q_{2}$$

where $\varphi_{i,j}$ is given by

(14) $$\begin{array}{cc} \varphi_{i,j}= & \log_{2}\left[\frac{\sqrt{b_{j}}\left(a_{i}+g_{i}\right)}{a_{i}\sqrt{\lambda_{1}+d_{j}\mu_{2}}+\sqrt{b_{j}\left(a_{i}+g_{i}\right)\left(\lambda_{1}+c_{i}\mu_{1}\right)-a_{i}g_{i}\left(\lambda_{1}+d_{j}\mu_{2}\right)}}\right]\\ & +\frac{{\left[\sqrt{b_{j}\left(a_{i}+g_{i}\right)\left(\lambda_{1}+c_{i}\mu_{1}\right)-a_{i}g_{i}\left(\lambda_{1}+d_{j}\mu_{2}\right)}+a_{i}\sqrt{\lambda_{1}+d_{j}\mu_{2}}\right]}^{2}}{b_{j}{\left(a_{i}+g_{i}\right)}^{2}}\\ & -\frac{1}{2}\left(\log_{2}e+1+\log_{2}\ln2\right) \end{array}$$

when $p_{s,i}^{⋆}>0$ and $b_{j}\left(\lambda_{1}+c_{i}\mu_{1}\right)>g_{i}\left(\lambda_{1}+d_{j}\mu_{2}\right)$ hold;

(15) $$\varphi_{i,j}=\log_{2}\left[\sqrt{\frac{g}{\lambda_{1}+c_{i}\mu_{1}}}\right]+\frac{\lambda_{1}+c_{i}\mu_{1}}{g}-\frac{1}{2}\left(\log_{2}e+1+\log_{2}\ln2\right)$$

when $p_{s,i}^{⋆}>0$ and $b_{j}\left(\lambda_{1}+c_{i}\mu_{1}\right)\leq g_{i}\left(\lambda_{1}+d_{j}\mu_{2}\right)$ hold; and $\varphi_{i,j}=0$ when $p_{s,i}^{⋆}=0$ holds. By comparing the two Equations (14) and (15), it is not difficult to find that as long as *x* > *y* is satisfied, the value of the relaying link is greater than that of the direct link. This again proves that the selection criteria are correct. Now, only the subcarrier pairing variables are left. Obviously, the problem (13) is a typical linear assignment problem, which has been solved in [26]. Finally, dual variables have to be searched iteratively to satisfy the power and interference threshold constraints.

## 4. Resource Allocation under Separate Power Constraints

If SS and SR suffer from separate power constraints, then the sum rate maximization problem is formulated by

(16) $$\begin{array}{cc} \max_{\left\{p_{s,i},p_{r,j},\rho_{i,j}\right\}} & \sum_{i=1}^{N}\sum_{j=1}^{N}\rho_{i,j}r_{i,j}\\ s.t. & \sum_{i=1}^{N}p_{s,i}\leq P_{S},\sum_{j=1}^{N}p_{r,j}\leq P_{R}\\ \; & \sum_{i=1}^{N}p_{s,i}c_{i}\leq Q_{1},\sum_{j=1}^{N}p_{r,j}d_{j}\leq Q_{2}\\ \; & \sum_{i=1}^{N}\rho_{i,j}=1,\sum_{j=1}^{N}\rho_{i,j}=1,\forall i,j \end{array}$$

where $P_{S}$ and $P_{R}$ are the peak power budgets at SS and SR, respectively.

### 4.1. Power Allocation

Similarly, the Lagrangian function of Equation (16) is constructed as

(17) $$\begin{array}{cc} L= & \sum_{i=1}^{N}\sum_{j=1}^{N}\rho_{i,j}r_{i,j}+\lambda_{1}\left[P_{S}-\sum_{i=1}^{N}p_{s,i}\right]+\lambda_{2}\left[P_{R}-\sum_{j=1}^{N}p_{r,j}\right]+\mu_{1}\left[Q_{1}-\sum_{i=1}^{N}p_{s,i}c_{i}\right]+\mu_{2}\left[Q_{2}-\sum_{j=1}^{N}p_{r,j}d_{j}\right]\\ = & \sum_{i=1}^{N}\sum_{j=1}^{N}\rho_{i,j}\left[r_{i,j}-\lambda_{1}p_{s,i}-\lambda_{2}p_{r,j}-\mu_{1}p_{s,i}c_{i}-\mu_{2}p_{r,j}d_{j}\right]+\lambda_{1}P_{S}+\lambda_{2}P_{R}+\mu_{1}Q_{1}+\mu_{2}Q_{2} \end{array}$$

According to the KKT rule, taking the partial derivative of *L* with respect to $p_{s,i}$ and $p_{r,j}$, we get

(18) $$\begin{array}{cc} \frac{\partial L}{\partial p_{s,i}}= & \frac{a_{i}b_{j}^{2}p_{r,j}^{2}+a_{i}^{2}g_{i}p_{s,i}^{2}+b_{j}^{2}g_{i}p_{r,j}^{2}+2a_{i}b_{j}g_{i}p_{s,i}p_{r,j}}{2\ln2\left(a_{i}p_{s,i}+b_{j}p_{r,j}\right)\left(a_{i}p_{s,i}+b_{j}p_{r,j}+a_{i}b_{j}p_{s,i}p_{r,j}+b_{j}g_{i}p_{s,i}p_{r,j}+a_{i}g_{i}p_{s,i}^{2}\right)}-\lambda_{1}-\mu_{1}c_{i}\\ = & 0 \end{array}$$

(19) $$\begin{array}{cc} \frac{\partial L}{\partial p_{r,j}}= & \frac{a_{i}^{2}b_{j}p_{s,i}^{2}}{2\ln2\left(a_{i}p_{s,i}+b_{j}p_{r,j}\right)\left(a_{i}p_{s,i}+b_{j}p_{r,j}+a_{i}b_{j}p_{s,i}p_{r,j}+b_{j}g_{i}p_{s,i}p_{r,j}+a_{i}g_{i}p_{s,i}^{2}\right)}-\lambda_{2}-\mu_{2}d_{j}\\ = & 0 \end{array}$$

After some algebraic operations, the optimal power allocation is given by

(20) $$\begin{array}{cc} p_{s,i}^{⋆}= & {\left[\frac{a_{i}b_{j}^{2}f_{i,j}^{2}+a_{i}^{2}g_{i}+2a_{i}b_{j}g_{i}f_{i,j}+b_{j}^{2}g_{i}f_{i,j}^{2}}{2\ln2\left(a_{i}+b_{j}f_{i,j}\right)\left(\lambda_{1}+c_{i}\mu_{1}\right)\left(b_{j}g_{i}f_{i,j}+a_{i}b_{j}f_{i,j}+a_{i}g_{i}\right)}-\frac{a_{i}+b_{j}f_{i,j}}{b_{j}g_{i}f_{i,j}+a_{i}b_{j}f_{i,j}+a_{i}g_{i}}\right]}^{+} \end{array}$$

(21) $$\begin{array}{cc} p_{r,j}^{⋆}= & f_{i,j}p_{s,i} \end{array}$$

where

(22) $$f_{i,j}=\left\{\begin{array}{cc} \frac{a_{i}\sqrt{\left(\lambda_{2}+d_{j}\mu_{2}\right)\left(a_{i}b_{j}\left(\lambda_{1}+c_{i}\mu_{1}\right)+b_{j}g_{i}\left(\lambda_{1}+c_{i}\mu_{1}\right)-a_{i}g_{i}\left(\lambda_{2}+d_{j}\mu_{2}\right)\right)}-a_{i}g_{i}\left(\lambda_{2}+d_{j}\mu_{2}\right)}{b_{j}\left(a_{i}+g_{i}\right)\left(\lambda_{2}+d_{j}\mu_{2}\right)} & \mathrm{if}b_{j}\left(\lambda_{1}+c_{i}\mu_{1}\right)\\ \; & >g_{i}\left(\lambda_{2}+d_{j}\mu_{2}\right)\\ 0 & \mathrm{otherwise} \end{array}\right.$$

In the separate power constraints, the decision criterion for whether to relay becomes $b_{j}\left(\lambda_{1}+c_{i}\mu_{1}\right)>g_{i}\left(\lambda_{2}+d_{j}\mu_{2}\right)$. If the condition $b_{j}\left(\lambda_{1}+c_{i}\mu_{1}\right)>g_{i}\left(\lambda_{2}+d_{j}\mu_{2}\right)$ holds, SS prefers to communicate with SD via the help of the relay station SR. Otherwise, direct transmission is more beneficial. Next, let’s look at an interesting special case. When there is only one subcarrier, the optimal power allocation is given by $p_{s}=\min\left(P_{S},Q_{1}/c\right)$ and $p_{r}=\min\left(P_{R},Q_{2}/d\right)$.

### 4.2. Subcarrier Pairing

Substituting the power variables into the *L*, we get

(23) $$L=\sum_{i=1}^{N}\sum_{j=1}^{N}\rho_{i,j}\varphi_{i,j}+\lambda_{1}P_{S}+\lambda_{2}P_{R}+\mu_{1}Q_{1}+\mu_{2}Q_{2}$$

where $\varphi_{i,j}$ is given by

(24) $$\begin{array}{cc} \varphi_{i,j}= & \log_{2}\left[\frac{\sqrt{b_{j}}\left(a_{i}+g_{i}\right)}{a_{i}\sqrt{\lambda_{2}+d_{j}\mu_{2}}+\sqrt{b_{j}\left(a_{i}+g_{i}\right)\left(\lambda_{1}+c_{i}\mu_{1}\right)-a_{i}g_{i}\left(\lambda_{2}+d_{j}\mu_{2}\right)}}\right]\\ & +\frac{{\left[\sqrt{b_{j}\left(a_{i}+g_{i}\right)\left(\lambda_{1}+c_{i}\mu_{1}\right)-a_{i}g_{i}\left(\lambda_{2}+d_{j}\mu_{2}\right)}+a_{i}\sqrt{\lambda_{2}+d_{j}\mu_{2}}\right]}^{2}}{b_{j}{\left(a_{i}+g_{i}\right)}^{2}}\\ & -\frac{1}{2}\left(\log_{2}e+1+\log_{2}\ln2\right) \end{array}$$

when $p_{s,i}^{⋆}>0$ and $b_{j}\left(\lambda_{1}+c_{i}\mu_{1}\right)>g_{i}\left(\lambda_{2}+d_{j}\mu_{2}\right)$ hold;

(25) $$\varphi_{i,j}=\log_{2}\left[\sqrt{\frac{g}{\lambda_{1}+c_{i}\mu_{1}}}\right]+\frac{\lambda_{1}+c_{i}\mu_{1}}{g}-\frac{1}{2}\left(\log_{2}e+1+\log_{2}\ln2\right)$$

when $p_{s,i}^{⋆}>0$ and $b_{j}\left(\lambda_{1}+c_{i}\mu_{1}\right)\leq g_{i}\left(\lambda_{2}+d_{j}\mu_{2}\right)$ hold; and $\varphi_{i,j}=0$ when $p_{s,i}^{⋆}=0$ holds. Similarly, the problem (23) is a typical linear assignment problem, which has been solved in [26]. Finally, dual variables have to be searched iteratively to satisfy the power and interference threshold constraints.

## 5. Simulation Results

Simulation results are provided in this section to prove our proposed optimization algorithm. Assume six tap channels taken from COST207 model for all links [27]. We define the average SNR as $P_{T}/N_{0}$. Furthermore, the transmission signal to noise ratio (SNR) of the primary user is assumed to be $q_{s,i}/N_{0}=0$ dB, ∀i. In addition, the number of subcarriers are set to be K=32. For simplicity, SS and SR share the same peak power $P_{S}=P_{R}=P_{T}/2$. We consider a common simulation layout, where the source, the relay, and the destination are deployed in a straight line and the relay is deployed at the midpoint unless otherwise specified. The path loss exponent is assumed 3.

**Figure 1:**
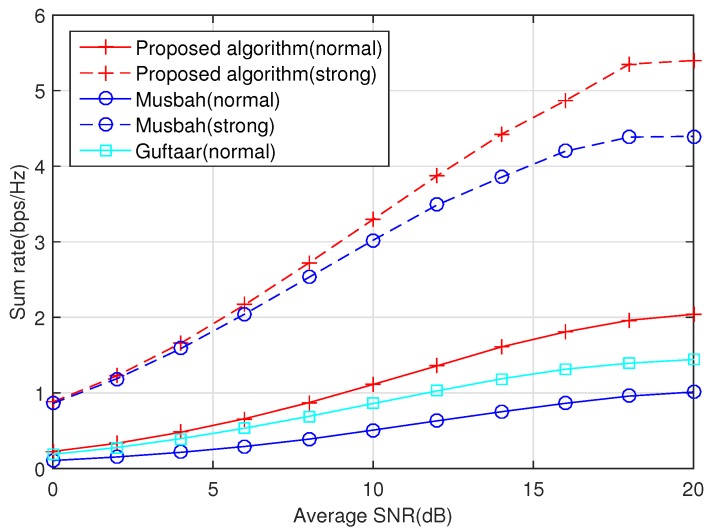
A comparison of different algorithms, where normal means E$\left(a_{i}\right)$ = E$\left(b_{i}\right)$ = E$\left(g_{i}\right)$ = 1 and strong means E$\left(a_{i}\right)$ = E$\left(b_{i}\right)$ = 1, E$\left(g_{i}\right)$ = 10.

Figure 1 compares the performance of our proposed algorithm with that of Musbah’s method [16] and Guftaar’s method [17], where normal means that all channel gains are normalized to unit one, while strong means that the channel quality of the direct link is 10 dB higher than that of the relaying link. It can be seen from Figure 1 that, when the channel quality of the direct link is good, the rate of Musbah’s exceeds that of Guftaar’s. Conversely, when the channel quality of direct link deteriorates, the rate of Musbah’s method drops sharply and is lower than that of Guftaar’s. This phenomenon fully shows that Musbah’s method focuses on the direct link instead of the relaying link, while Guftaar’s focuses on the relaying link instead of the direct link. However, our proposed algorithm outperforms the other two approaches in the same environment because we can adaptively access the two links according to the decision criteria:

Next, we prepare to compare the performance of the following suboptimal algorithms. (1) Fixed subcarrier: The subcarriers received and forwarded by the relay are the same, i.e., $\rho_{i,j}=1$ for i=j and $\rho_{i,j}=0$ otherwise. This method is equivalent to power allocation without subcarrier pairing in case with the proposed algorithm; (2) Equal power: Equal power is distributed across all subcarriers at secondary users. Specifically, the powers have to be respectively adjusted as $p_{s,i}=\min\left(P_{T}/\left(2K\right),Q_{1}/\sum_{i}\sum_{j}\rho_{i,j}c_{i}\right)$ and $p_{r,j}=\min\left(P_{T}/\left(2K\right),Q_{2}/\sum_{i}\sum_{j}\rho_{i,j}d_{j}\right)$ under total power constraint and $p_{s,i}=\min\left(P_{1}/K,Q_{1}/\sum_{i}\sum_{j}\rho_{i,j}c_{i}\right)$ and $p_{r,j}=\min\left(P_{2}/K,Q_{2}/\sum_{i}\sum_{j}\rho_{i,j}d_{j}\right)$ under separate power constraints due to the interference condition of the primary user. (4) Fixed subcarrier and equal power (FSEP): The subcarrier pairing is fixed as in case (1) and power is evenly distributed as in case (2).

**Figure 2:**
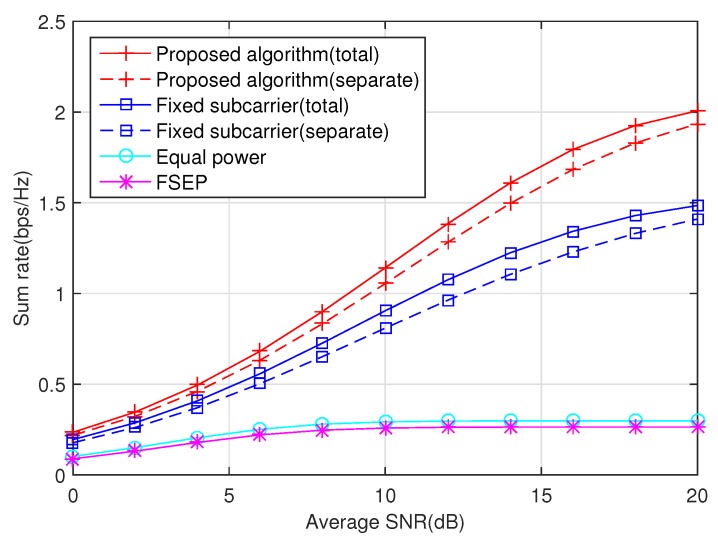
The impact of the average SNR on the sum rate under the interference threshold $Q_{1}=Q_{2}=0$ dB.

The impact of the average SNR on the sum rate is showed in Figure 2. We observe that the joint algorithm exhibits a substantial increase in promoting the sum rate of the secondary users at all SNR values. For example, at a target rate 1 bps/Hz, the optimal algorithm can save about 2 dB SNR in contrast to a fixed subcarrier case. Even the equal power case and FSEP case can not reach this target rate in this simulation experiment. Moreover, by comparing fixed subcarrier case and equal power case, we find that the power allocation is more efficient than subcarrier pairing in the aspect of sum rate promotion. Part of the fact is that, in the equal power scheme, the subcarrier pairing is not optimal due to the existence of interference constraints.

**Figure 3:**
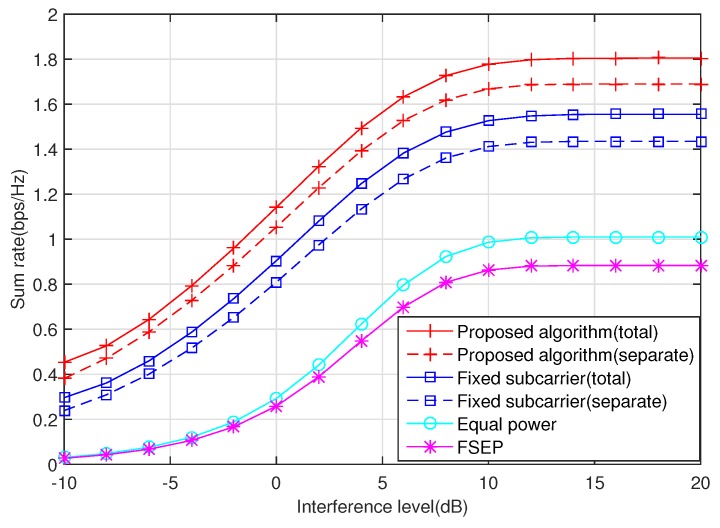
The effect of the interference constraint level on the sum rate, where the average SNR is 10 dB.

Next, the effect of the interference constraint level $Q_{1}$ on the sum rate is plotted in Figure 3, where $Q_{1}=Q_{2}$. The higher the level of interference the primary user can tolerate, the higher the transmission rate of the secondary user is because more power can be consumed by the secondary user as long as the interference to the primary user is lower than the threshold value. Obviously, when the interference level is relatively low, the sum rate of the secondary user is dominated by strict protection criteria imposed by the primary user. In this case, the secondary user does not use the full transmission power. When the interference level is high, the sum rate of the secondary user is governed by the total power budget. In this case, the interference caused by the secondary user to the primary user does not reach the maximum tolerable interference level. Thus, in the high interference threshold regime, the sum rate remains unchanged. In fact, at a low interference level, the power constraint is inactive while at a high interference level, the interference constraint condition is inactive.

**Figure 4:**
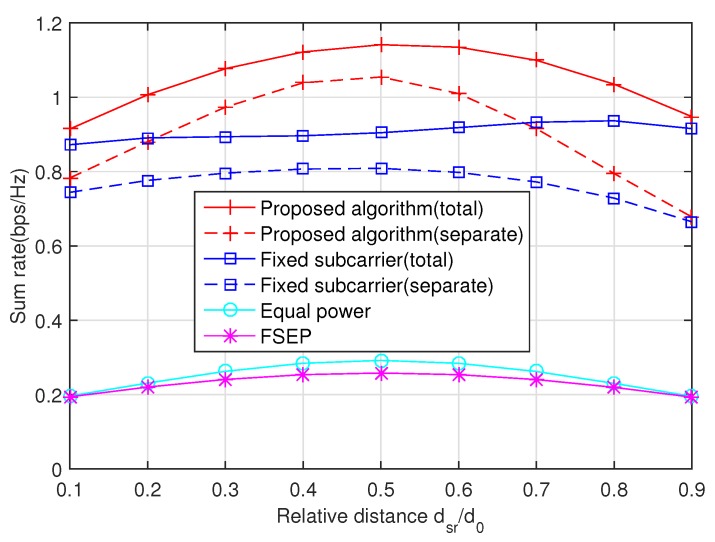
The impact of relay position on the sum rate, where the average SNR is 10 dB, $d_{sr}$, and $d_{0}$ are the distances between SS and SR and between SS and SD, respectively.

Then, the impact of relay position on the sum rate is drawn in Figure 4, where the relay moves between the source and destination. The key observation is that the relay prefers to stay at midway. This phenomenon shows that the relay can effectively help the source node in data transmission, especially in the scenario where the destination node is far from the source node.

**Figure 5:**
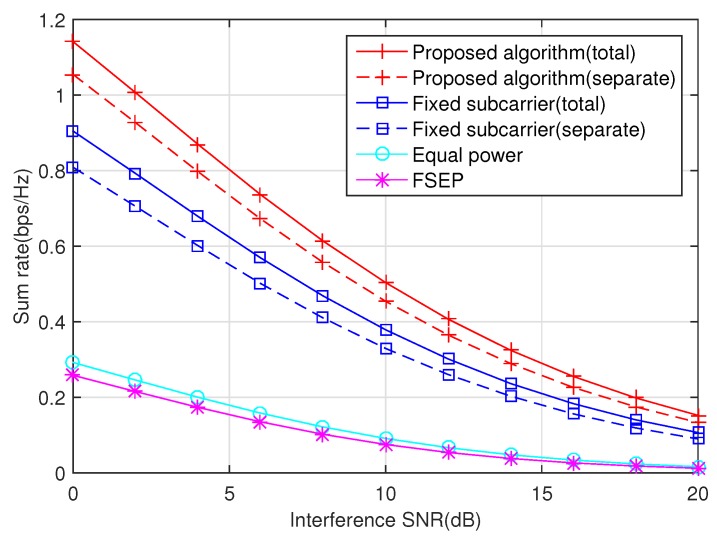
The influence of the interference SNR on the sum rate, $P_{T}/N_{0}$ = 10 dB.

The influence of the average interference SNR $q_{s,i}/N_{0}$ of the primary user on the sum rate of the secondary user is illustrated in Figure 5. The transmission power of the primary user is an interference from the perspective of the secondary user. Thus, the sum rate decreases when the primary user increases its transmission power.

**Figure 6:**
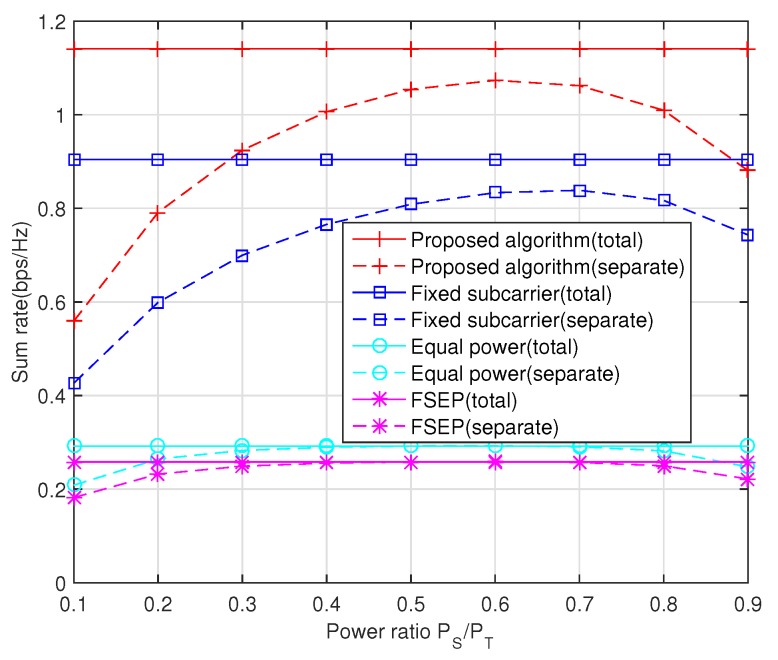
The impact of the power ratio $P_{S}/P_{T}$ on the sum rate, $P_{T}/N_{0}$=10 dB.

Finally, the impact of the power ratio $P_{S}/P_{T}$ on the sum rate is described in Figure 6, where $P_{R}=P_{T}-P_{S}$ is always maintained to ensure fairness under the separate power constraint. When $P_{S}$ and $P_{R}$ are subject to the total power constraint, they can adaptively adjust to find the optimal power allocation scheme. Thus, the sum rate of the total power case, which can be regarded as the upper bound of separate power case, always keeps flat in all ratios. Moreover, from a separate case, more power should be provided to the source node because sometimes the relay may turn off its transceiver when direct link is more efficient than relaying link.

## 6. Conclusions

This paper has studied resource allocation problems in AF cognitive relaying OFDM networks. Two typical problems are fully investigated. One is the sum rate maximization of the secondary user under a total power constraint and the other is subject to individual power constraints. The rate performance of the secondary user is different from that of the traditional cooperative networks due to the interference constraints of the primary user. The simulation results show that the secondary user’s rate suffers a significant loss even with a large power budget when the interference threshold is relatively low. In future, we will consider precoding and cross layer optimization issues in cognitive relay networks. 

## Figures and Tables

**Table 1:** A comparison of different works.

References	Cognitive	OFDM	Relaying	Direct	Power	Subcarrier
	Radio		Protocol	Link	Allocation	Pairing
[6]			AF		*√*	
[7]			AF	*√*		
[8]	*√*	*√*	AF		*√*	*√*
[9]	*√*	*√*	AF		*√*	*√*
[10]	*√*	*√*	AF		*√*	*√*
[11]	*√*	*√*	AF and DF	*√*	*√*	
[12]	*√*	*√*	DF	*√*		
[13]	*√*	*√*	DF	*√*	*√*	*√*
[14]	*√*	*√*		*√*	*√*	
[15]	*√*	*√*		*√*	*√*	
[16]	*√*	*√*		*√*	*√*	
[17]	*√*	*√*	AF		*√*	*√*
[18]	*√*		AF	*√*		
[19]	*√*	*√*	AF	*√*		
[20]	*√*		AF	*√*	*√*	
[21]	*√*	*√*	AF	*√*	*√*	
[22]	*√*		DF	*√*	*√*	
Our paper	*√*	*√*	AF	*√*	*√*	*√*
